# A novel deep learning model for a computed tomography diagnosis of coronary plaque erosion

**DOI:** 10.1038/s41598-023-50483-9

**Published:** 2023-12-27

**Authors:** Sangjoon Park, Haruhito Yuki, Takayuki Niida, Keishi Suzuki, Daisuke Kinoshita, Iris McNulty, Alexander Broersen, Jouke Dijkstra, Hang Lee, Tsunekazu Kakuta, Jong Chul Ye, Ik-Kyung Jang

**Affiliations:** 1 Department of Bio and Brain Engineering, Korea Advanced Institute of Science and Technology, Daejeon, South Korea; 2 Cardiology Division, Massachusetts General Hospital, Harvard Medical School, 55 Fruit Street, GRB 800, Boston, MA 02114 USA; 3 Department of Radiology, Division of Image Processing, Leiden University Medical Center, Leiden, the Netherlands; 4 Biostatistics Center, Massachusetts General Hospital, Harvard Medical School, Boston, MA n USA; 5 Department of Cardiology, Tsuchiura Kyodo General Hospital, Tsuchiura, Ibaraki Japan; 6 Kim Jaechul Graduate School of Artificial Intelligence, Department of Mathematical Sciences, Korea Advanced Institute of Science and Technology, 291 Daehak-Ro, Daejeon, 34141 South Korea

**Keywords:** Interventional cardiology, Translational research, Acute coronary syndromes, Vascular diseases

## Abstract

Patients with acute coronary syndromes caused by plaque erosion might be managed conservatively without stenting. Currently, the diagnosis of plaque erosion requires an invasive imaging procedure. We sought to develop a deep learning (DL) model that enables an accurate diagnosis of plaque erosion using coronary computed tomography angiography (CTA). A total of 532 CTA scans from 395 patients were used to develop a DL model: 426 CTA scans from 316 patients for training and internal validation, and 106 separate scans from 79 patients for validation. *Momentum Distillation-enhanced Composite Transformer Attention (MD-CTA)*, a novel DL model that can effectively process the entire set of CTA scans to diagnose plaque erosion, was developed. The novel DL model, compared to the convolution neural network, showed significantly improved AUC (0.899 [0.841–0.957] vs. 0.724 [0.622–0.826]), sensitivity (87.1 [70.2–96.4] vs. 71.0 [52.0–85.8]), and specificity (85.3 [75.3–92.4] vs. 68.0 [56.2–78.3]), respectively, for the patient-level prediction. Similar results were obtained at the slice-level prediction AUC (0.897 [0.890–0.904] vs. 0.757 [0.744–0.770]), sensitivity (82.2 [79.8–84.3] vs. 68.9 [66.2–71.6]), and specificity (80.1 [79.1–81.0] vs. 67.3 [66.3–68.4]), respectively. This newly developed DL model enables an accurate CT diagnosis of plaque erosion, which might enable cardiologists to provide tailored therapy without invasive procedures.

**Clinical Trial Registration:** http://www.clinicaltrials.gov, NCT04523194.

## Introduction

Acute coronary syndromes (ACS) are the most common cause of death worldwide^1^. Previous reports have revealed that plaque rupture was the underlying mechanism in the majority of cases. However, an alternative pathology, plaque erosion, has been gaining attention as recent in vivo studies demonstrated that erosion is responsible for 25–60% of cases^2,3^. In current clinical practice, ACS patients are uniformly treated with stenting regardless of the underlying pathology^4^. Although not recommended by the current guidelines, recent studies reported that conservative management without coronary stenting might be an option for ACS patients caused by plaque erosion^5^.

Currently, a diagnosis of plaque erosion can only be made by intracoronary optical coherence tomography (OCT), which requires an invasive procedure and expertise in image interpretation. The use of coronary computed tomography angiography (CTA) has been increasing exponentially over the last several years. Several studies investigated specific features of plaque erosion on CTA^6–8^. However, due to limited resolution, coronary CTA lacks the ability to make an accurate diagnosis of plaque erosion in which structural changes are subtle.

In recent years, deep learning (DL) has been applied to various medical fields, including medical imaging. Recent works have reported the DL application on automated coronary CTA analyses ranging from segmentation to classification, but the targets for identification were confined to easily discernible findings such as stenosis^9^ or calcification^10^, and the diagnosis of challenging entities such as plaque erosion has never been reported. Moreover, explicitly training the model for diagnosis of plaque erosion has never been possible, as the number of patients who underwent coronary CTA paired with concurrent OCT was limited.

We aimed to develop a DL model to make an accurate diagnosis of plaque erosion non-invasively with coronary CTA. To achieve this aim, we devised the “*Momentum Distillation-enhanced Composite Transformer Attention* (MD-CTA)” model that can incorporate the information from the entire scans, emulating the reading process of the human experts who look at the slices back-and-forth to make an accurate diagnosis and utilizing the modality-specific self-supervised learning strategy to enhance the performance.

## Methods

### Study population

Patients with ACS (non-ST-segment elevation myocardial infarction [NSTEMI] or unstable angina pectoris [UAP]) or stable angina pectoris (SAP) who underwent both coronary CTA and OCT prior to percutaneous coronary intervention (PCI) were included from the database, “Massachusetts General Hospital (Massachusetts, USA) and Tsuchiura Kyodo General Hospital (TKGH) (Ibaraki, Japan) Coronary Imaging Collaboration” (NCT04523194). NSTEMI and UAP were diagnosed using American Heart Association /American College of Cardiology guidelines^11^. NSTEMI was defined as ischemic symptoms in the absence of ST-segment elevation on the electrocardiogram with elevated cardiac biomarkers. UAP was defined as having newly developed or accelerating ischemic symptoms on exertion or rest angina within 2 weeks without biomarker release. SAP was defined as chest pain on exertion without changes in frequency, intensity, and duration of symptoms in the previous 4 weeks and/or a positive stress test. The culprit lesion was defined as the site of PCI, the tightest lesion, or the lesion with evidence of recent plaque disruption on coronary angiogram. In cases of multivessel PCI, the lesions with the highest degree of stenosis as assessed on angiogram were chosen as the culprit lesion, and all the culprit lesions were confirmed by OCT. A total of 596 patients (ACS: 300, SAP: 296) who presented between January 2011 and September 2022 were included. Among ACS patients, 14 patients were excluded for calcified plaque, 1 for spontaneous coronary artery dissection, 2 for coronary spasm, and 1 for myocardial infarction with non-obstructive coronary artery. In addition, 15 patients were excluded for poor image quality, 2 for in-stent restenosis, 2 for no OCT images before PCI, 5 for culprit lesions located in the left main, 1 for culprit lesion located in the diagonal branch, and 1 for staged PCI. Among SAP patients, 139 patients who had vessel segments greater than 10 mm in length with no plaque as assessed by angiography and CTA imaging were included in the non-erosion group. Thus, 256 ACS (113 with plaque erosion, 143 with plaque rupture) and 139 SAP patients were included in the final analysis (Supplementary Fig. S1A). The Massachusetts General Hospital and TKGH Coronary Imaging Collaboration study was approved by the Institutional Review Boards at Massachusetts General Hospital and Tsuchiura Kyodo General Hospital. Written informed consent for enrollment in the TKGH’s institutional database for potential future investigations was provided by all participants. The study protocol conforms to the ethical guidelines of the Declaration of Helsinki.

### Coronary CTA acquisition and analysis

Coronary CTA image acquisition was performed using a 320-slice CT scanner (Aquilion ONE; Canon Medical Systems Corporation, Otawara, Tochigi, Japan) in accordance with the Society of Cardiovascular Computed Tomography guidelines^12^. Oral and/or intravenous beta-blockers were administered if a patient’s resting heart rate was > 65 bpm. Sublingual nitroglycerin (0.3 or 0.6 mg) was administered immediately before CT scanning. Coronary CTA images were acquired with the following scan protocol: tube voltage of 120 kVp, tube current of 50 to 750 mA, the gantry rotation speed of 350 ms per rotation, and field matrix of 512 × 512, and scan slice thickness of 0.5 mm. Acquisition of CT data and the electrocardiography (ECG) trace were automatically started as soon as the signal density level in the ascending aorta reached a predefined threshold of 150 Hounsfield units. Images were acquired after a bolus injection of 30 to 60 mL of contrast media (iopamidol, 370 mg iodine/mL, Bayer Yakuhin, Ltd., Osaka, Japan) at a rate of 3 to 6 mL/s, using prospective ECG-triggering or retrospective ECG-gating with automatic tube current modulation. All scans were performed during a single breath-hold. Images were reconstructed at a window centered at 75% of the R-R interval to coincide with left ventricular diastasis. All intervals between CTA slices were 0.25 mm. The coronary CTA datasets were analyzed on a cardiac workstation with dedicated analysis software (Qangio CT RE 3.1, Medis, Leiden, the Netherlands), as previously reported^13^. Analysis began with the automatic detection of the coronary arteries followed by the segmentation of luminal and outer vessel boundaries. If needed, manual adjustments of the vessel centerline and boundaries were performed.

### OCT analysis

OCT examination was performed using either a frequency-domain (C7/C8, OCT Intravascular Imaging System, St. Jude Medical, St. Paul, Minnesota) or a time-domain (M2/M3 Cardiology Imaging Systems, LightLab Imaging Inc., Westford, Massachusetts) OCT system. The images were analyzed by three independent investigators who were blinded to patients’ data, using an offline review workstation (St. Jude Medical). Qualitative and quantitative analyses were performed using previously established criteria^14^ by independent investigators blinded to the clinical, angiographic, and laboratory data.

### Cross-correlation with CTA and OCT images

As previously reported, the matching of OCT and CTA images was performed using an offline algorithm (Matcher version 2.1 Leiden, the Netherlands)^15^. In the first step, the OCT images were mapped onto the CT image along the vessel centerline using anatomical landmarks. In the second step, the individual OCT images were translated and rotated to fit best on the CT image, using the vessel center and landmarks for orientation. The algorithm also corrected for deviations in the OCT pullback speed by using interpolation between landmarks.

Among 256 ACS patients, the diagnosis of plaque erosion (n = 113) or rupture (n = 143) on OCT was used as the ground truth and the site on the CTA image that matched the culprit plaque on the OCT image was determined to be the culprit lesion. In addition, 276 CTA scans without plaques detected by OCT and/or angiography and CTA images were chosen as the scans with no plaque. Thus, a total of 532 CTA scans were included in the final analysis (113 CTA scans with plaque erosion, 143 with plaque rupture, and 276 scans with no plaque [Supplementary Fig. S1B]).

For the development and validation of the deep learning model, CTA images in digital imaging and communications in medicine (DICOM) format and their corresponding labels were transferred to the Bio-Imaging, Signal Processing, and Learning laboratory at the Korea Advanced Institute of Science and Technology after anonymization.

Among 395 patients (532 CTA scans), the data were divided into non-overlapping patient subsets, training and cross-validation datasets containing 316 patients (426 CTA scans) for model development and tuning, and the test set containing 79 patients (106 CTA scans) for final performance evaluation (Supplementary Methods and Supplementary Figs. S1C and S2) The disease prevalence of the non-plaque erosion class is 33.3% in the training set and 37.3% in the test set.

### Development and evaluation of the deep learning algorithm

As we aimed to develop a DL model that can discriminate between plaque erosion and other entities, we divided labels into two classes: plaque erosion and non-plaque erosion. In the non-plaque erosion class, plaque rupture, as well as the other images without significant lesions were included.

To make an accurate diagnosis, we had to take the entire collection of CTA images into consideration. Thus, we designed a vision transformer (ViT)-based model tailored to the data structure of CTA, dubbed the MD-CTA model. Unlike most contemporary medical AI models that lack the ability to incorporate the information of the entire volume, we utilized the transformer model (16) tailored for sequential data structure. Specifically, we simultaneously optimized the spatial transformer that extracts the information within a single slice and the sequence transformer that incorporates the extracted information of all slices to produce the final outcome. We trained the model using both the slice-level and patient-level annotations to enable the model to learn the location of the lesion of interest as well as the label classes. We also implemented the standard convolutional neural network (CNN) based model for comparison with the same design and settings as the proposed DL model (Supplementary Methods, Supplementary Table S1, and Supplementary Figs. S3, S4). We performed the internal five-fold cross-validation to get the best hyperparameter as well as evaluate the model performance. The model is visualized via the attention weights of the spatial and sequence transformers (Supplementary Methods).

A reader study was performed to evaluate the clinical utility of the DL model as an assisting tool as well as to compare the performances with experienced cardiologists. The definition of the experienced cardiologist is provided in Supplementary Methods. In the first round, the performance of the DL model for the test set was compared with that of experienced cardiologists. Then, in the second round, the prediction results by the DL model along with the corresponding CTA scans were provided to the readers to evaluate whether the diagnostic performances were improved with the model’s assistance (Supplementary Methods).

### Statistical analysis

Categorical data are presented as counts and percentages, and are compared using the chi-squared test or Fisher exact test, as appropriate. Continuous variables have been shown as mean ± SD or median (25th to 75th percentiles), as appropriate, depending on the normality of distribution. Per-lesion data were analyzed using the generalized estimating equations with a logit link for the binary variables to consider the potential clustering of multiple plaques in a single patient. Between-group differences in continuous variables were compared using the Student t-test or Mann–Whitney U test, as appropriate. A P value < 0.05 was considered statistically significant.

The model performance was evaluated with the area under the receiver-operating-characteristic curves (AUC), and the sensitivities, specificities, accuracy, positive predictive values (PPVs), and negative predictive values (NPVs) were calculated for the detailed analysis. To estimate the false alarms by the model, the false-positive rate (FPR) and false-negative rate (FNR) were calculated. The 95% confidence intervals (Cis) were calculated by DeLong’s method for AUC, and “exact” Clopper-Pearson confidence intervals for sensitivity, specificity, accuracy, and false estimates. Likewise, the standard logit confidence intervals were used to estimate the 95% Cis of the predictive values.

All analyses were performed with SPSS 28.0 (version 28.0 for Windows; SPSS, Inc., Chicago, Illinois) and Python Scikit-learn package (Python version 3.8, Scikit-learn version 1.1.2, http://scikit-learn.org/).

## Results

### Study population

For model development and internal validation, we used a total of 532 CTA scans from 395 patients. Patients were randomly divided into non-overlapping subsets, training and cross-validation datasets for model development and tuning (426 scans from 316 patients), and the test set for final performance evaluation (containing 106 scans from 79 patients) (Supplementary Fig. S1C).

Detailed patient characteristics are summarized in Table 1. Other than a higher prevalence of diabetes mellitus in the training dataset than in the test dataset (109 [34.5%] vs. 18 [22.8%], p = 0.018), no differences were observed in patient characteristics, medications, and laboratory data between the two datasets. The subset of patients with ACS showed the same pattern (Diabetes: 78 [38.6%] vs. 11 [20.4%], p = 0.013) (Supplementary Table S2). When the location of the culprit lesion and underlying pathology were compared between training and test datasets, no significant difference was found between the two groups (Table 2). Among patients with ACS, there were no significant differences in qualitative and quantitative OCT analyses between training and test datasets (Supplementary Table S2). In addition, when minimum lumen area measured by OCT and area stenosis measured by CTA were calculated in patients with plaque erosion or rupture, there were no significant differences between the two groups (Supplementary Table S3).

**Table 1:** Patient characteristics in the training versus test datasets.

Variables		Overall patients (n = 395)		P value
		Training dataset (n = 316, 80.0%)	Test dataset (n = 79, 20.0%)	
Age (years)		66.5 (58.3–74.0)	68.5 (59.0–76.0)	0.893
Male		257 (81.3)	66 (83.5)	0.648
Hypertension		188 (59.5)	48 (60.8)	0.690
Dyslipidemia		135 (42.7)	40 (50.6)	0.371
Diabetes mellitus		109 (34.5)	18 (22.8)	0.018
Current smoking		93 (29.4)	24 (30.4)	0.951
Renal insufficiency		102 (32.3)	22 (27.8)	0.448
Ejection fraction (%)		63 (56–67)	64 (55–68)	0.618
NSTE-ACS	NSTEMI	161 (50.9)	37 (46.8)	0.513
	UAP	41 (13.0)	17 (21.5)	0.055
SAP		114 (36.1)	25 (31.6)	0.461
Previous MI		40 (12.7)	6 (7.6)	0.210
Previous PCI		49 (15.5)	10 (12.7)	0.519
Previous CABG		4 (1.3)	0 (0.0)	0.315
Medication on admission				
Aspirin		85 (26.9)	22 (27.8)	0.609
DAPT		60 (19.0)	16 (20.3)	0.782
ACE-I/ARB		100 (31.6)	33 (41.8)	0.160
Statin		111 (35.1)	25 (31.6)	0.348
Β-blocker		130 (41.1)	35 (44.3)	0.902
Laboratory data				
WBC (count/µL)		6660 (5380–8738)	6965 (5565–8735)	0.901
Triglycerides (mg/dL)		114 (82–183)	124 (84–197)	0.399
Total cholesterol (mg/dL)		185 ± 2.6	192 ± 5.7	0.450
LDL cholesterol (mg/dL)		111 (88–137)	117 (92–138)	0.671
HDL cholesterol (mg/dL)		46 (40–56)	46 (41–56)	0.944
HbA1c (%)		5.9 (5.5–6.8)	5.9 (5.5–6.6)	0.355
eGFR (mL/min/1.73m^2^)		72.9 (61.2–83.3)	72.5 (62.9–84.4)	0.212

Values are mean ± SD, n (%), or median (25th-75th percentile).

*ACE-I* angiotensin-converting enzyme inhibitor, *ARB* angiotensin II receptor blocker, *CABG* coronary artery bypass graft, *DAPT* dual anti-platelet therapy, *eGFR* estimated glomerular filtration rate, *HbA1c* hemoglobin A1c, *HDL* high-density lipoprotein, *LDL* low-density lipoprotein, *MI* myocardial infarction, *NSTE-ACS* non-ST-segment elevation acute coronary syndromes, *NSTEMI* non-ST-segment elevation myocardial infarction, *PCI* percutaneous coronary intervention: *SAP* stable angina pectoris, *UAP* unstable angina pectoris, *WBC* white blood cell.

**Table 2:** Coronary CTA scan lesion characteristics in the training versus test datasets.

Variables	Overall CTA scans (n = 532)		P value
	CTA scans in training dataset (n = 426, 80.1%)	CTA scans in test dataset (n = 106, 19.9%)	
Lesion location			
RCA	135 (31.7)	37 (34.9)	0.762
LAD	184 (43.2)	43 (40.6)	
LCX	107 (25.1)	26 (24.5)	
Pathology			
Plaque erosion	90 (21.1)	23 (21.7)	0.904
Plaque rupture	112 (26.3)	31 (29.2)	0.577
No plaque	224 (52.6)	52 (49.1)	0.598

Values are n (%).

*CTA* computed tomography angiography, *LAD* left anterior descending artery, *LCX* left circumflex artery, *RCA* right coronary artery.

### Diagnostic performances of the deep learning model

**Figure 1:**
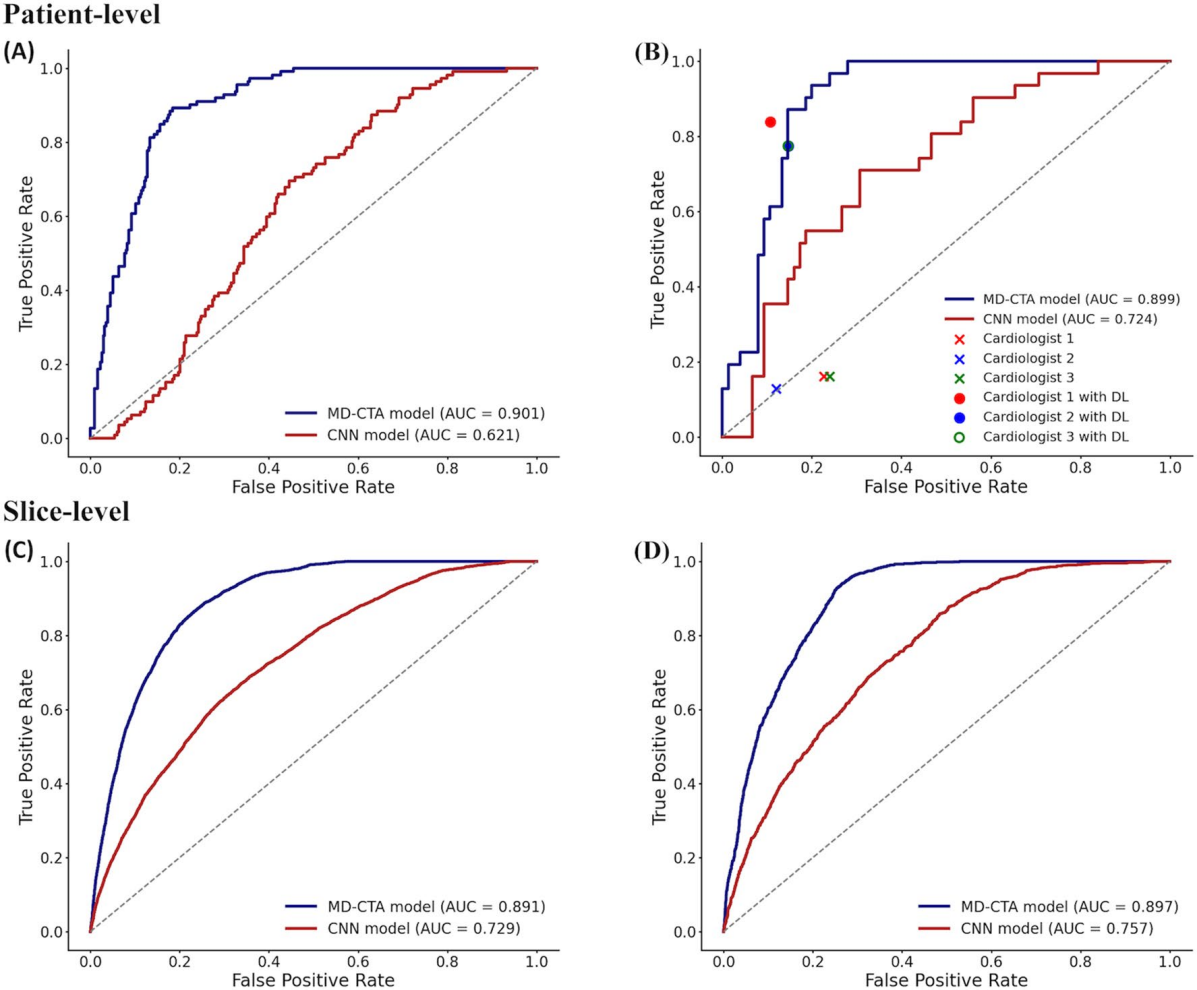
Diagnostic accuracy of the deep learning (DL) models for patient-level (**A**,**B**) and slice-level (**C**,**D**) predictions. Diagnostic performance of the deep learning models at the patient level in the five-fold cross-validation (**A**), in the test set validation (**B**), and at the slice level in the five-fold cross-validation (**C**), in the test set validation (**D**). *AUC* area under the curve, *CNN* convolutional neural network, *MD-CTA* momentum distillation-enhanced composite transformer attention.

Patient-level prediction performances for plaque erosion are shown in Fig. 1A,B and Table 3. In the five-fold cross-validation, the MD-CTA model showed diagnostic performance with an AUC of 0.901 (0.873–0.930), sensitivity of 81.2 (72.8–88.0), and specificity of 86.6 (82.4–90.2), all of which were significantly higher than those of the CNN model with an AUC of 0.621 (0.567–0.675), sensitivity of 59.8 (50.1–69.0), and specificity of 60.2 (54.5–65.7). Similarly, in the test set validation, the AUC, sensitivity, and specificity of the DL model were 0.899 (0.841–0.957), 87.1 (70.2–96.4), and 85.3 (75.3–92.4), respectively, higher than those of 0.724 (0.622–0.826), 71.0 (52.0–85.8), and 68.0 (56.2–78.3) of the CNN model. In both five-fold cross-validation and test set validation, the NPVs were higher than 90.0%, but PPVs were relatively low (68.4 and 71.1, respectively) due to the smaller number of positives.

**Table 3:** Performances of the deep learning models for patient-level diagnosis.

	AUC (95% CI)	Sensitivity (%) (95% CI)	Specificity (%) (95% CI)	PPV (95% CI)	NPV (95% CI)	FPR (%) (95% CI)	FNR (%) (95% CI)
Five-fold cross-validation							
MD-CTA model	0.901 (0.873–0.930)	81.2 (72.8–88.0)	86.6 (82.4–90.2)	68.4 (61.7–74.4)	92.8 (89.8–95.0)	13.4 (9.8–17.6)	18.8 (12.0–27.2)
CNN model	0.621 (0.567–0.675)	59.8 (50.1–69.0)	60.2 (54.5–65.7)	34.9 (30.4–39.7)	80.8 (76.7–84.3)	39.8 (34.3–45.5)	40.2 (31.0–49.9)
Test set validation							
MD-CTA model	0.899 (0.841–0.957)	87.1 (70.2–96.4)	85.3 (75.3–92.4)	71.1 (58.3–81.2)	94.1 (86.5–97.6)	14.7 (7.6–24.7)	12.9 (3.6–29.8)
CNN model	0.724 (0.622–0.826)	71.0 (52.0–85.8)	68.0 (56.2–78.3)	47.8 (38.1–57.8)	85.0 (76.2–90.9)	32.0 (21.7–43.8)	29.0 (14.2–48.0)

*AUC* area under the curve, *CI* confidence interval, *CNN* convolutional neural network, *FNR* false-negative rate, *FPR* false-positive rate, *MD-CTA* momentum distillation-enhanced composite transformer attention, *NPV* negative predictive value, *PPV* positive predictive value.

Slice-level prediction performances are provided in Fig. 1C,D and Table 4. In the five-fold cross-validation, the MD-CTA model provided the diagnostic performances with an AUC of 0.891 (0.887–0.895), sensitivity of 82.9 (81.7–84.0), and specificity of 80.0 (79.5–80.5), while the CNN model showed an AUC of 0.729 (0.722–0.737), sensitivity of 66.2 (64.8–67.6), and specificity of 66.8 (66.3–67.4). Likewise, in the test set validation, the MD-CTA model’s AUC, sensitivity, specificity, and accuracy were 0.897 (0.890–0.904), 82.2 (79.8–84.3), and 80.1 (79.1–81.0), while those of the CNN model were 0.757 (0.744–0.770), 68.9 (66.2–71.6), and 67.3 (66.3–68.4), respectively. The NPVs for the slice level prediction were over 90.0% in both five-fold cross-validation and the test set validation, while the PPVs were relatively low, attributed to the imbalance between positives and negatives.

**Table 4:** Performances of the deep learning models for slice-level diagnosis.

	AUC (95% CI)	Sensitivity (%) (95% CI)	Specificity (%) (95% CI)	PPV (95% CI)	NPV (95% CI)	FPR (%) (95% CI)	FNR (%) (95% CI)
Five-fold cross-validation							
MD-CTA model	0.891 (0.887–0.895)	82.9 (81.7–84.0)	80.0 (79.5–80.5)	38.5 (37.9–39.1)	96.9 (96.7–97.1)	20.0 (19.5–20.5)	17.1 (16.0–18.3)
CNN model	0.729 (0.722–0.737)	66.2 (64.8–67.6)	66.8 (66.3–67.4)	23.2 (22.7–23.7)	92.9 (92.6–93.2)	33.2 (32.6–33.7)	33.8 (32.4–35.2)
Test set validation							
MD-CTA model	0.897 (0.890–0.904)	82.2 (79.8–84.3)	80.1 (79.1–81.0)	39.3 (38.0–40.5)	96.6 (96.2–97.0)	19.9 (19.0–80.9)	17.8 (15.7–20.2)
CNN model	0.757 (0.744–0.770)	68.9 (66.2–71.6)	67.3 (66.3–68.4)	24.9 (23.9–25.8)	93.3 (92.7–93.8)	32.7 (31.6–33.7)	31.1 (28.4–33.8)

*AUC* area under the curve, *CI* confidence interval, *CNN* convolutional neural network, *FNR* false-negative rate, *FPR* false-positive rate, *MD-CTA* momentum distillation-enhanced composite transformer attention, *NPV* negative predictive value, *PPV* positive predictive value.

The model performances evaluated exclusively on the ACS patients are provided in the Supplementary Results, Supplementary Table S4. In the test set validation, the Cohen’s kappa coefficient was 0.679 between the model and the ground truth labels. When the two key components, the composite transformer attention and the modality-specific self-supervised pre-training, were not used together, the performance of the vanilla ViT model’s performances were sub-optimal (Supplementary Results and Supplementary Table S5). The diagnostic performance of the same model for culprit lesion is presented in the Supplementary Result and Supplementary Table S6.

### Analysis of the false estimates

Tables 3 and 4 show the results of the analysis of the false estimates. In the five-fold cross-validation, the FPR and FNR of the MD-CTA model were 13.4 (9.8–17.6) and 18.8 (12.0–27.2) for the patient-level diagnosis, and 20.0 (19.5–20.5) and 17.1 (16.0–18.3) for the slice-level diagnosis, which was lower than the CNN model. In the test set validation, the FPR and FNR of the MD-CTA model were 14.7 (7.6–24.7) and 12.9 (3.6–29.8) for the patient-level, and 19.9 (19.0–80.9) and 17.8 (15.7–20.2) for the slice-level diagnoses, providing lower false estimates than the CNN model. More detailed information on false estimates is provided in the Supplementary Results and Supplementary Fig. S5.

### Model interpretability results

**Figure 2:**
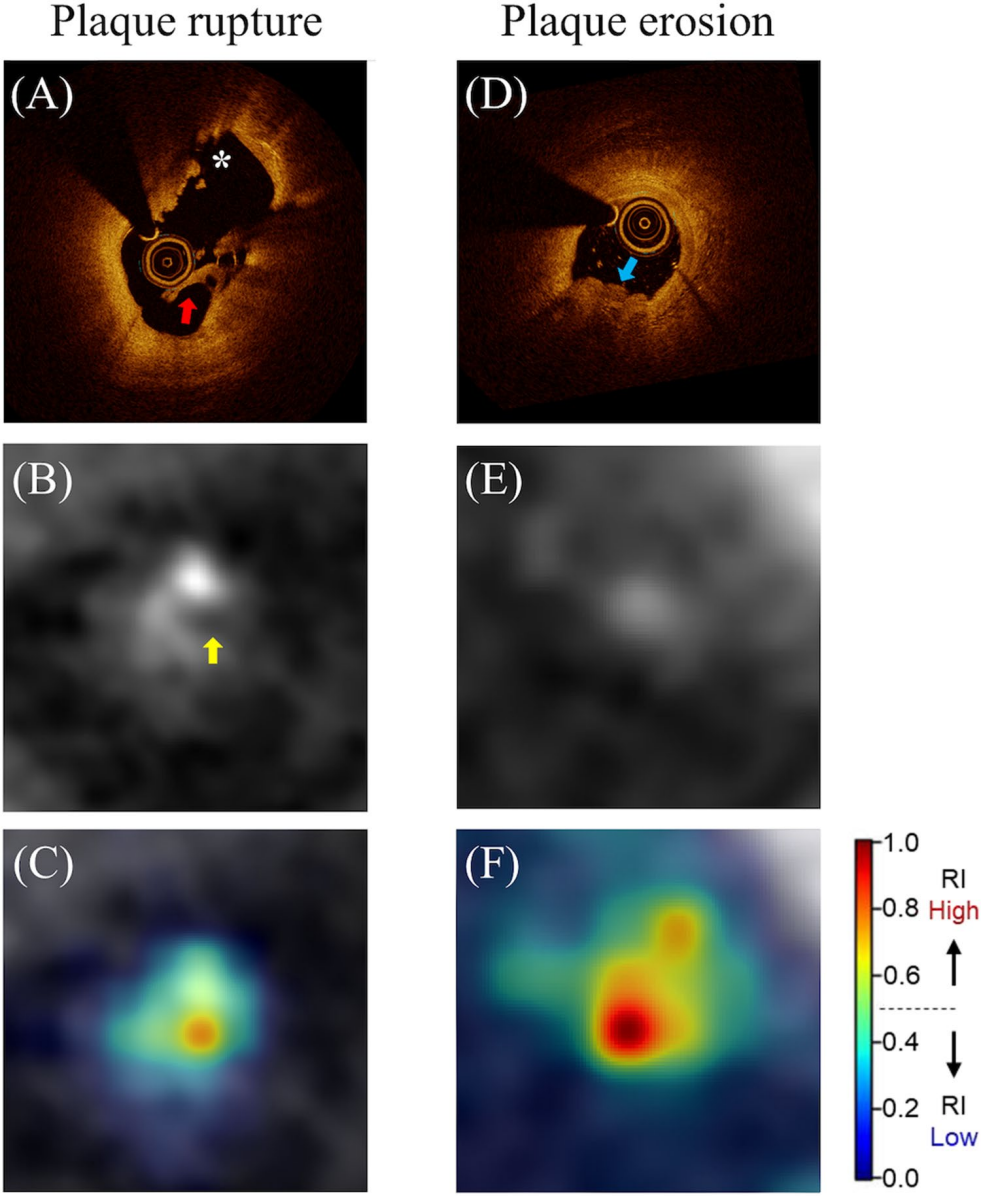
Plaque rupture and plaque erosion as seen on OCT, CTA, and CTA enhanced by DL model. Representative images of each label are shown. (**A**) shows an OCT image of plaque rupture. Plaque rupture is characterized by the presence of fibrous cap discontinuity with a cavity formation (asterisks) within the plaque. (**A**) also shows the residual ruptured cap (red arrow). (**B**,**C**) show CTA images of the corresponding site. (**B**) shows the ruptured cap (yellow arrow) protruding into the vessel lumen at the same site observed by OCT. (**C**) shows that the DL model attends on the ruptured cap and cavity. (**D**) shows an OCT image of plaque erosion. Definite plaque erosion is characterized by the presence of attached thrombus (blue arrow) overlying an intact and visualized plaque. (**E**,**F**) show CTA images of the corresponding site. (**E**) shows a small lumen surrounded by plaque without a cavity. (**F**) shows that the DL model attends on the site of stenosis without evidence of a cavity. In panels (**C**) and (**F**), the visualized model attention represents the relative importance as determined by the DL model for each specific image, with values normalized to a range between 0 and 1 for the images under consideration. *CTA* computed tomography angiography, *DL* deep learning, *OCT* optical coherence tomography, *RI* relative importance.

We visualized the attention of the slice-level and sequence-level transformer, which reflect the model’s attention within the slice and between the slices, respectively. The relative importance estimated by the model has been normalized between 0 (low) and 1 (high), and this estimated relative importance is visualized in accordance with a scale bar, as depicted in Fig. 2. As provided in the representative cases in Fig. 2, Supplementary Fig. S6, and Supplementary Videos S1 and S2, the DL model paid attention accurately to the lesion location compared to the ground truth annotation at the patient level. Within a single frame, the suspected culprit lesion was well localized by the model attention, suggesting that the model can identify the clinically important features within the given frame.

### Reader study comparing the model performance with the experienced cardiologists

In the first round of the reader study, the performances of the MD-CTA model were compared with the experienced cardiologists as shown in Table 5. The model outperformed the expert readers in all diagnostic performance metrics, and the superiority of the model was most prominent for the sensitivity (87.1% in DL model vs. 16.1% in reader 1, 12.9% in reader 2, and 16.1% in reader 3). The second round of the reader study was performed to evaluate whether the model can be used as an assisting tool to improve the diagnostic performance of the human reader. When given the model’s prediction results for the probability and location of the plaque erosion, the diagnostic performances of the human readers markedly improved, especially for the sensitivity, increasing from 16.1% to 83.9% in reader 1, from 12.9% to 77.4% in reader 2, and from 16.1% to 77.4% in reader 3.

**Table 5:** Results of the reader study to validate the clinical utility of the deep learning model.

	Sensitivity (%) (95% CI)	Specificity (%) (95% CI)	PPV (95% CI)	NPV (95% CI)	FPR (%) (95% CI)	FNR (%) (95% CI)
Novel DL model	87.1 (70.2–96.4)	85.3 (75.3–92.4)	71.1 (58.3–81.2)	94.1 (86.5–97.6)	14.7 (7.6–24.7)	12.9 (3.6–29.8)
Before DL model assistance						
Reader 1	16.1 (5.5–33.7)	77.3 (66.2–86.2)	22.7 (10.6–42.1)	69.1 (64.7–73.1)	22.7 (13.8–33.8)	83.9 (66.3–94.5)
Reader 2	12.9 (3.6–29.8)	88.0 (78.4–94.4)	30.8 (12.9–57.2)	71.0 (67.6–74.1)	12.0 (5.6–21.6)	87.1 (70.2–96.4)
Reader 3	16.1 (5.5–33.7)	76.0 (64.8–85.1)	21.7 (10.2–40.5)	68.7 (64.2–72.8)	24.0 (14.9–35.2)	83.9 (66.3–94.5)
After DL model assistance						
Reader 1	83.9 (66.3–94.6)	89.3 (80.1–95.3)	76.5 (62.4–86.4)	93.1 (85.7–96.8)	10.7 (4.7–9.9)	16.1 (5.4–33.7)
Reader 2	77.4 (58.9–90.4)	85.3 (75.3–92.4)	68.6 (55.0–79.6)	90.1 (82.6–94.6)	14.7 (7.6–24.7)	22.6 (9.6–41.1)
Reader 3	77.4 (58.9–90.4)	85.3 (75.3–92.4)	68.6 (55.0–79.6)	90.1 (82.6–94.6)	14.7 (7.6–24.7)	22.6 (9.6–41.1)

*CI* confidence interval, *DL* deep learning, *FNR* false-negative rate, *FPR* false-positive rate, *NPV* negative predictive value, *PPV* positive predictive value.

## Discussion

To the best of our knowledge, this is the first report showing that the automated diagnosis of plaque erosion is possible with a non-invasive coronary CTA using a novel DL algorithm. To achieve this goal, we have developed the MD-CTA model, which is able to leverage composite transformer attentions to incorporate the information and relationships between the coronary CTA slices, emulating the reading process of a human expert, and we enhanced the model’s performance with modality-specific self-supervised pre-training. The five-fold cross-validation and the test set validation results have shown that the DL model can diagnose plaque erosion solely from the CTA images, attaining a clinically useful level of diagnostic performance. Our model outperformed the experienced cardiologists, and when used as an assisting tool, the diagnostic performances of cardiologists were markedly improved.

Recent efforts to apply artificial intelligence to coronary artery imaging such as OCT, CTA, and IVUS have been made. However, most works were devoted to dense prediction and quantification like plaque segmentation^16,17^, or primarily focused on easily discernible abnormalities, for instance, thin-cap fibroatheroma^18^; only a minority of works have reported the DL application for the end-to-end diagnosis of specific findings. Although we have previously reported novel DL model for the diagnosis of plaque erosion, intravascular imaging with OCT was required^19^, thus, the use of the algorithm was restricted to the catheterization laboratory.

**Figure 3:**
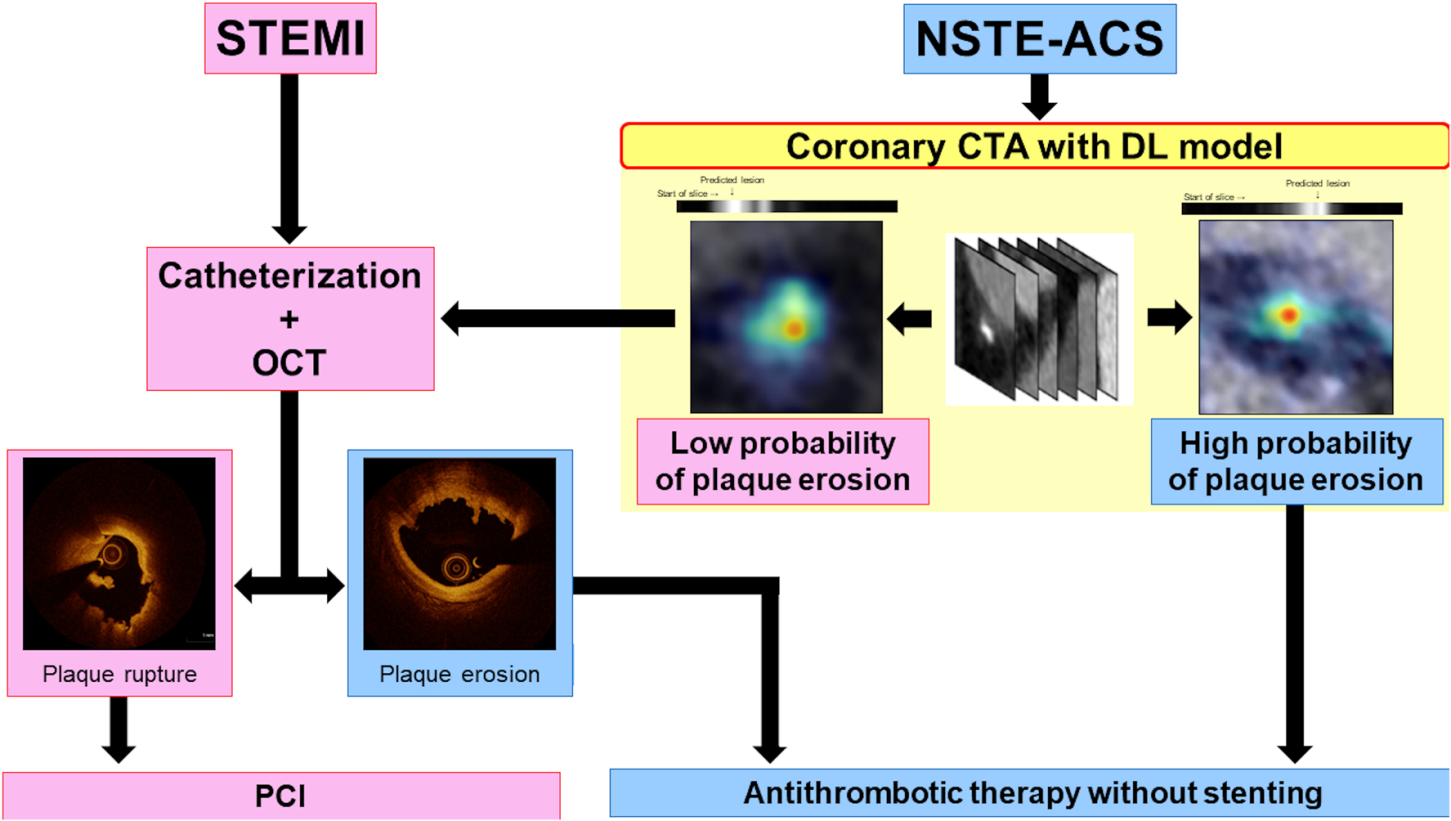
Potential Future Approach for Evaluation and Management of Patients With ACS. Patients with STEMI would undergo emergency catheterization. If plaque rupture is confirmed, the culprit lesion would be treated with stenting. If OCT demonstrated plaque erosion with preserved lumen, antithrombotic therapy without stenting could be considered. Patients with NSTE-ACS would undergo noninvasive coronary CTA with DL model after stabilization. If there is high probability of plaque erosion and preserved lumen, antithrombotic therapy without stenting could be considered. *CTA* computed tomography angiography, *DL* deep learning; *NSTE-ACS* non–ST-segment elevation acute coronary syndromes, *OCT* optical coherence tomography, *PCI* percutaneous coronary intervention, *STEMI* ST-segment elevation myocardial infarction.

Plaque erosion, which is responsible for up to 50% of patients with non-ST-segment elevation (NSTE)-ACS^2^, is characterized by an intact fibrous cap, preserved vascular structure, and platelet-rich thrombus. Thrombus in plaque erosion is attributed to apoptosis or denudation of superficial endothelial cells as opposed to fibrous cap disruption and creation of a cavity inside a plaque in plaque rupture. Previous studies have demonstrated that ACS patients with plaque erosion have fewer cardiovascular risk factors, less atherosclerotic burden, and lower frequency of complex lesions, less multivessel coronary artery disease, and higher prevalence of close proximity to a bifurcation than those with plaque rupture^20–22^. In addition, on OCT images, patients with plaque erosion have smaller reference vessel diameter, lower prevalence of calcification and thrombus in culprit lesions^20^, and lower prevalence of macrophage accumulation, microvessels, and spotty calcium in non-culprit lesions^23^ than those with plaque rupture. These findings might suggest that plaque erosion is associated with lower levels of pan-vascular vulnerability and exhibits rather subtle structural changes at the microscopic level^24^. If the aforementioned microscopic structural changes could be identified by using deep learning, plaque erosion can be diagnosed by these specific findings, rather be diagnosed by excluding plaque rupture, as it currently stands. Since patients with NSTE-ACS can usually be stabilized with medical therapy and preliminary data suggest that conservative management might be an option for ACS patients caused by plaque erosion^5,25^, we thought if we could make a diagnosis of plaque erosion by using CTA, this subset of patients might be able to be managed without invasive procedures (Fig. 3). The challenge with CTA is its capability to detect the subtle structural changes that occur in plaque erosion due to its lower resolution. We successfully surmounted this conundrum by leveraging the following approaches. First, we utilized a unique database comprised of paired coronary CTA and OCT images obtained simultaneously from the same subject. This approach enabled the model to learn from superior supervision regarding the presence and location of plaque erosion than CTA alone. As a result, the trained DL model could detect subtle changes in CT attenuation that might not be visible to the human eye. Had we built a model for diagnosing plaque erosion using the dataset lacking paired OCT images, the model’s performance would have been restricted to learning only from the lesions detectable by human experts in CTA images. Secondly, we integrated a novel design of composite transformer attention along with a self-supervised learning method to endow the model with a comprehensive understanding of the structural features of the CTA volume. Our approach resulted in a remarkable improvement in diagnostic performance compared to conventional CNN models. Specifically, in slices that were ambiguous and perplexing, the proposed MD-CTA model utilized a composite of intra-slice and inter-slice attention to provide a more precise diagnosis. In recent years, there have been more than 800,000 patients with myocardial infarction in the United States per year^26^ and NSTEMI has recently become the most frequent type of MI (NSTEMI increased from 52.8% in 2002 to 68.6% in 2011)^27^. In patients with NSTEMI, plaque erosion is the underlying pathology in up to 75% of cases^2^. Thus, the potential number of patients who might benefit from this new approach is enormous.

### Study limitations

Our study has several limitations. First, this was a retrospective analysis of patients who underwent both CTA and OCT prior to PCI. Therefore, selection bias cannot be excluded because patients without significant stenosis on CTA might have been excluded from invasive procedures such as coronary angiography or OCT. Second, the test set validation was performed only for the randomly split subset from the single data source. We adopted this approach as it was not possible to conduct the external validation in another institution since multi-modality imaging data with an adequate number of patients were not available elsewhere. To alleviate concerns for the generalizability, we did not use any vendor-specific pre- or post-processing, and the raw Hounsfield Unit values were used as the input of the model after simple normalization between 0–1. Furthermore, we employed two methods to improve the generalizability, namely transfer learning from pre-trained models on general domain data and domain-specific self-supervised learning. Without these methods, the performance was found to be compromised, suggesting the possibility of overfitting. Third, instead of histological ground truth, the concurrent OCT images that have higher resolution were leveraged as the gold standard. This approach was adopted since it was impossible to obtain histological diagnosis in living patients. This approach has been widely adopted in developing the DL model for medical image analysis when histological validation is not feasible^28–30^. Fourth, less common ACS pathologies such as a calcified plaque, spontaneous coronary dissection, and intraplaque acksedge were excluded. Fifth, although the prevalence of disease in non-plaque erosion was not low (34.1%), the possibility of the falsely high sensitivity of the MD-CTA model could not be completely ruled out. Sixth the diagnostic accuracy of the model tends to be affected by the quality of the image, for instance, severely calcified plaque or severe luminal narrowing lowered the accuracy of the diagnosis. Of note, plaque erosion, compared to plaque rupture, in general has a larger lumen. Seventh, the performance of the model slightly decreased when evaluated only on ACS patients. Furthermore, while the model demonstrates excellent performance in the overall diagnosis of plaque erosion versus non-plaque erosion, it shows a reduced performance in differentiating between plaque erosion and rupture. Nonetheless, our MD-CTA model clearly outperforms the CNN model (Supplementary Table S7). This suggests that our model may be utilized in clinical applications for purposes such as a screening tool for anomaly, based on its excellent detection capabilities for culprit lesions. Eighth, although previous small studies suggested that conservative management without coronary stenting might be an option for ACS patients with plaque erosion, data from prospective large-scale randomized trials are currently not available. Thus, further studies are warranted prior to the implementation of this approach into daily clinical practice. Finally, although we used the unique and well-curated dataset consisting of paired coronary CTA and OCT, the size of the dataset may still be small. Although large-scale studies with clinical outcomes would be helpful, combined pre-procedure CTA and intracoronary imaging in the same patients with ACS would be practically challenging.

## Conclusions

The MD-CTA model, specifically designed for coronary CTA and trained with the paired coronary CTA and OCT database, appears promising in identifying atherosclerotic plaque erosion using non-invasive coronary CTA images and significantly outperformed experienced cardiologists. Further research is needed to validate the usefulness of this novel model in clinical practice.

### Supplementary Information

Supplementary Information.

Supplementary Video 1.

Supplementary Video 2.

## Data Availability

The datasets used and/or analysed during the current study are available from the corresponding author upon reasonable request.

## Abbreviations

ACS: Acute coronary syndromes
AUC: Area under the receiver-operating characteristic curve
CI: Confidence interval
CNN: Convolutional neural network
CTA: Computed tomography angiography
DL: Deep learning
NSTE-ACS: Non-ST-segment elevation acute coronary syndromes
NSTEMI: Non-ST-segment elevation myocardial infarction
OCT: Optical coherence tomography
PCI: Percutaneous coronary intervention
SAP: Stable angina pectoris
UAP: Unstable angina pectoris
