# Supplementary material for: A novel deep learning model for a computed tomography diagnosis of coronary plaque erosion
Source: Sci Rep. 2023 Dec 27;13:22992. doi: 10.1038/s41598-023-50483-9 (PMC10752868; doi:10.1038/s41598-023-50483-9)
Supplement: Supplementary file 1 — Supplementary Information. [file 41598_2023_50483_MOESM1_ESM.docx]

**Supplementary Information**

**Supplementary Appendix**

Deep learning enabled the diagnosis of plaque erosion with coronary computed tomography angiography in patients with acute coronary syndromes.

**Contents**

**Supplementary Methods**

Coronary CTA Acquisition and Analysis

Definition of the training, validation, and test datasets

Development of the deep learning model

Reader Study details

**Supplementary Results**

Diagnostic performances evaluated on ACS patients

Ablation studies on the proposed model’s key components

Detailed analyses of the false estimates

Detection performances for culprit lesions

**Supplementary Tables**

Supplementary Table S1. Summary of hyperparameters of the deep learning model

Supplementary Table S2. Comparison of baseline characteristics in ACS patients between training and test datasets

Supplementary Table S3. Comparison of MLA and intervals between OCT slices in ACS patients between plaque erosion and rupture

Supplementary Table S4. Comparison of diagnostic performances evaluated on ACS patients

Supplementary Table S5. Ablation studies on the key components of the Momentum Distillation-enhanced Composite Transformer Attention (MD-CTA) model

Supplementary Table S6. Slice-level detection performances of the culprit lesion of the deep learning models

**Supplementary Figures**

Supplementary Figure S1. Flow diagram of the study

Supplementary Figure S2. Details of the dataset division for the study

Supplementary Figure S3. Architecture of the Momentum Distillation-enhanced Composite Transformer Attention (MD-CTA) model

Supplementary Figure S4. Training strategy of the Momentum Distillation-enhanced Composite Transformer Attention (MD-CTA) model

Supplementary Figure S5. Confusion matrices of the model

Supplementary Figure S6. Representative images of plaque rupture and plaque erosion as seen on OCT, CTA, and CTA enhanced by DL model

**Supplementary Videos**

Supplementary Video S1. Representative images of plaque rupture on OCT

Supplementary Video S2. Representative images of plaque erosion on OCT

**Supplementary Methods**

*Coronary CTA Acquisition and Analysis*

All patients included in the current study had been stabilized by medical therapy prior to CTA. If the patient's condition was deemed unstable even under medical therapy, urgent PCI was performed without CTA, and they were excluded from this study. The median time from CTA to OCT was 3.0 hours (2.0-25.0).

*Definition of the training, validation, and test datasets*

In deep learning, the entire data corpus is divided into subsets: the training, validation, and test datasets. The training dataset is the corpus that model sees and learns the pattern of data. It is primarily used to fit the model parameters, for instance, by training on the data and labels through supervised learning methods using optimization methods like gradient descent ^[1]^. As the training step proceeds, the model eventually gets overfitted to the training dataset, meaning that it is exorbitantly biased to the data pattern seen during the training, and therefore the generalization performance may be exacerbated. A subset of data, the validation dataset, is used to ameliorate the bias while tuning the hyperparameters of the model. It is for the evaluation of a given model but used for frequent evaluation by deep learning developers to fine-tune the model hyperparameters. To further reduce the bias caused by the subset division, k-fold cross-validation (e.g. five-fold cross-validation) was performed to provide coherent evaluation results for the data used for the model development and tuning. The test dataset, which should be separated prior to the model development and should only be used once the model is complete, provides the gold standard for the evaluation of results on an independent set of data. As it is used to estimate the model’s performance in a real-world application, the external dataset collected from an independent institution with different acquisition settings is often used.

*Development of the deep learning model*

For decades, convolutional neural network (CNN) based models have been widely used as the standard model architecture for medical images as well as computer vision, thanks to their outstanding performance with the convolution operation ^[2]^. More recently, however, Transformer-based models ^[3]^, which were originally invented for natural language processing (NLP), have overtaken the CNN-based models with their scaling properties and powerful self-attention mechanism. Vision Transformer (ViT) ^[4]^, relying on pure self-attention, is the first successful attempt that surpassed the state-of-the-art CNN-based model without using the convolution operation at all, unleashing the era of transformer-based vision models. Besides its outstanding scalability that can benefit more from large model and dataset sizes, recent research has proven that the ViT possesses useful properties desired in the domain of medical imaging. Recent studies have shown that the ViT has the shape-biased property ^[5]^, which means that the model makes a decision, concentrating more on the shape of the object rather than the background noise or other confounders. Moreover, it was reported that the well-trained ViT model has a flatter loss landscape compared to the CNN-based model ^[6]^, allowing generalizability for many tasks. Finally, the knowledge distillation-based self-supervised learning strategy, an autodidactic learning approach where the student model learns from the teacher model’s prediction in place of the label, was shown to be especially effective for the ViT models ^[7]^.

Inspired by these works, here we introduced a novel ViT-based deep learning model for coronary computed tomography angiography (CTA) image processing and analysis, dubbed the Momentum Distillation-enhanced Composite Transformer Attention (MD-CTA) model. The proposed MD-CTA consists of two key components: composite transformer attention and momentum distillation. For the first component, we used transformer attention in two ways. First, the pure ViT model equipped with the self-attention within each slice, named *spatial transformer*, is used to encode the CTA slices into a more compact feature vector with a dimension of 384. The *sequential transformer* subsequently gets the feature vectors of all slices and outputs the transformed feature incorporating the information of the entire CTA scans. Slice-level and the patient-level prediction results are obtained from the transformed feature with the simple multi-layer perception and the average pooling operation, respectively. As it has been reported that multi-task learning may improve the performance of deep learning models, we have simultaneously optimized the model with two learning objectives from two different tasks, the slice-level prediction and the patient-level prediction as described in Supplemental Figure 5.

To overcome the vulnerability to overfitting caused by training our model on a relatively small dataset, we adopted approaches that have been developed in recent deep learning research to achieve comparable performance to models trained on large-scale data, even with a limited amount of data ^[8-10]^. First, we initialized the spatial transformer using the pre-trained weights on ImageNet, to perform transfer learning from well-trained general domain data. Additionally, we trained the model to learn domain-specific knowledge through the use of momentum distillation-enhanced self-supervised pre-training, as illustrated in Supplemental Figure 4. Inspired by prior work on *distillation with no labels* in computer vision ^[7]^, we adapted this method to the sequential structure of the CTA scan. Specifically, we constructed two identical models as teacher and student, and cropped the CTA volume into a longer and smaller sequence. The longer sequence was input to the teacher, while the shorter sequence was input to the student. By allowing the student model to match the prediction of the teacher model with less information (the shorter sequence), the model can learn the knowledge about the imaging modality without any handcrafted supervision.

We used the ViT-B/16 model pre-trained on ImageNet ^[7]^ as the spatial transformer, and the transformer equipped with 12 layers and 12 attention heads as the sequential encoder. Considering the complexity of the model, the ResNet-50 model was used as the standard CNN model for comparison.

The model was developed on the ubuntu system installed on Intel Core i7-11700K and Nvidia RTX 3090 graphic processing unit (GPU), which took approximately half a day for the training. We evaluated the model in the ubuntu environment with Intel Core i7-9700K and Nvidia RTX 3090 GPU.

*Reader Study details*

The reader study was performed to investigate the model’s utility as an aiding tool as well as to compare its performance to experienced cardiologists. The experienced cardiologist was defined as the one who had more than 8 years of experience ^[11]^ and had been supervised by a senior cardiologist with expertise in CTA interpretation. To this aim, we performed the reader study twice. First, the anonymized 106 CTA scans in the test set were given to three cardiologists along with an answer sheet to complete. The cardiologists were blinded to clinical information and OCT findings for a fair comparison with the model. The performance comparison was conducted by comparing their performances at this time with the model’s performances. In the second round, the test set was randomly shuffled again and given to the readers along with the model’s prediction, after a four week washout period to prevent the performance improvement from the recollection. In this round, we aimed to investigate whether giving the model’s prediction for a given CTA scan could improve the sensitivity, specificity, and accuracy of the experienced cardiologist’s coronary CTA reading.

**Supplementary Results**

*Diagnostic performances evaluated on ACS patients*

Supplemental Table 4 presents the results of our model's performance evaluation exclusively on the subset of patients with ACS in the test set. At the patient-level, the MD-CTA model achieved a sensitivity and specificity of 83.9 (66.3-94.6) and 70.4 (49.8-86.3), respectively, and these demonstrated a substantial improvement not only compared to the CNN model but also to human readers. Similarly, at the slice-level, the MD-CTA model exhibited a sensitivity and specificity of 74.5 (71.9-77.0) and 74.7 (73.4-76.1), which substantially outperformed the CNN model.

*Ablation studies on the model’s key components*

Supplemental Table 5 shows the results of the ablation studies for the key components of the MD-CTA model. As shown in the table, not performing transfer learning from the well-trained weights on the general domain resulted in significant degradation in performance. Compared with the vanilla ViT model with only the spatial attention or the ViT with sequential attention to integrating the information of the entire slices to make a decision, the proposed method adopting the modality-specific self-supervised pre-training with momentum distillation provided overall better performance in both patient-level and slice-level diagnosis, implying that the two key components are crucial for the optimal diagnostic performance for coronary CTA.

*Detailed analyses of the false estimates*

All 35 cases with false positives were attributed to missed small ruptures and mild irregularity of the lumen contour. In 11 cases of false positives, there was severe calcification in two and the other 9 had relatively severe stenosis. Out of 4 cases of false negatives, a large red thrombus was misdiagnosed as plaque rupture in 2, a side branch was misdiagnosed in 1 case, while a small thrombus attached to the lumen was missed by the DL model in 1 case.

*Detection performances for culprit lesions*

Supplemental Table 6 provides the results of evaluating the MD-CTA model's performance for identifying the location of culprit lesions at the slice level. The model demonstrated excellent performance, outperforming the CNN model, with an AUC, sensitivity, and specificity of 0.935 (0.932-0.938), 85.8 (85.4-86.2), and 85.5 (84.6-86.3), respectively, in the five-fold cross-validation. Similarly, in the test set validation, the MD-CTA model exhibited high detection performance, with an AUC, sensitivity, and specificity of 0.929 (0.924-0.934), 85.4 (84.5-86.2), and 83.3 (81.5-84.9), respectively, and provided significantly better diagnostic performance than the CNN model.

**Supplementary Table S1. Summary of hyperparameters of the deep learning model**

| **Hyperparameters** | **Value** |
| --- | --- |
| Input image size | 224 × 224 |
| Batch size | 1 |
| Learning rate | 0.00001 |
| Learning rate scheduler | WarmupCosine |
| Optimizer | AdamW |
| Pre-training epochs | 20 |
| Epochs | 30 |
| Weight decay coefficient | 0.0001 |

**Supplementary Table S2. Comparison of baseline characteristics in ACS patients between training and test datasets**

| Variables | ACS patients (n=256) | | P Value |
| --- | --- | --- | --- |
|  | **Training Dataset (n=202, 78.9%)** | **Test Dataset (n=54, 21.1%)** |  |
| Age (years) | 64.5 (54.3-71.8) | 60.0 (52.0-71.0) | 0.891 |
| Male | 164 (81.2) | 45 (83.3) | 0.718 |
| Hypertension | 134 (66.3) | 35 (64.8) | 0.858 |
| Dyslipidemia | 97 (48.0) | 25 (46.3) | 0.839 |
| Diabetes mellitus | 78 (38.6) | 11 (20.4) | **0.013** |
| Current smoking | 75 (37.1) | 19 (35.2) | 0.879 |
| Renal insufficiency | 43 (21.3) | 12 (22.2) | 0.882 |
| Ejection fraction (%) | 61 (52-65) | 63 (49-65) | 0.749 |
| NSTEMI | 161 (79.7) | 37 (68.5) | 0.081 |
| UAP | 41 (20.3) | 17 (31.5) | 0.081 |
| Previous MI | 16 (7.9) | 3 (5.6) | 0.556 |
| Previous PCI | 17 (8.4) | 4 (7.4) | 0.810 |
| Previous CABG | 4 (2.0) | 0 (0.0) | 0.297 |
| Medication on admission |  |  |  |
| Aspirin | 36 (17.8) | 9 (16.7) | 0.700 |
| DAPT | 16 (7.9) | 3 (5.6) | 0.486 |
| ACE-I / ARB | 84 (41.6) | 29 (53.7) | 0.104 |
| Statin | 56 (27.7) | 10 (18.5) | 0.172 |
| Β-blocker | 98 (48.5) | 28 (51.9) | 0.643 |
| Laboratory data |  |  |  |
| WBC (count/µL) | 7370 (5918-9835) | 8140 (6375-10020) | 0.919 |
| Triglycerides (mg/dL) | 119 (73-167) | 163 (72-202) | 0.420 |
| Total cholesterol (mg/dL) | 193±5.9 | 204±13.3 | 0.250 |
| LDL cholesterol (mg/dL) | 122±5.1 | 126±11.5 | 0.485 |
| HDL cholesterol (mg/dL) | 49±1.6 | 49±3.3 | 0.533 |
| Glucose (mg/dL) | 116 (100-153) | 113 (107-131) | 0.286 |
| HbA1c (%) | 5.8 (5.4-6.6) | 5.6 (5.3-5.8) | 0.127 |
| eGFR (mL/min/1.73m^2^) | 75.9 (66.3-84.9) | 80.3 (72.5-88.7) | 0.418 |
| Lesion location |  |  | 0.797 |
| RCA | 50 (24.8) | 14 (25.9) |  |
| LAD | 110 (54.5) | 31 (57.4) |  |
| LCX | 42 (20.8) | 9 (16.7) |  |
| Pathology |  |  |  |
| Plaque erosion | 90 (44.6) | 23 (42.6) | 0.796 |
| Plaque rupture | 112 (55.4) | 31 (57.4) | 0.796 |
| OCT findings |  |  |  |
| Lipid-rich plaque | 171 (84.7) | 48 (88.9) | 0.432 |
| TCFA | 84 (41.6) | 24 (44.4) | 0.705 |
| Macrophage | 158 (78.2) | 48 (88.9) | 0.079 |
| Microvessels | 96 (47.5) | 25 (46.3) | 0.872 |
| Cholesterol crystal | 58 (28.7) | 14 (25.9) | 0.686 |
| Layered plaque | 101 (50.0) | 26 (48.1) | 0.809 |
| Calcifications | 98 (48.5) | 24 (44.4) | 0.595 |
| Thrombus | 136 (67.3) | 37 (68.5) | 0.868 |
| Minimum fibro cap thickness (μm) | 70 (60-110) | 63 (51.5-106.5) | 0.736 |
| MLA (mm^2^) | 1.01 (0.69-1.18) | 1.05 (0.77-1.81) | 0.462 |
| Plaque length (mm) | 11.7 (9.2-15.0) | 12.2 (11.3-15.2) | 0.199 |
| Max lipid arc (°) | 239.3 (203.5-306.6) | 226.7 (185.8-311.5) | 0.811 |
| Mean lipid arc (°) | 203.6±6.5 | 201.5±12.8 | 0.924 |
| Lipid index (°mm) | 1557.7 (996.6-2570.3) | 1460.1 (1181.1-2211.0) | 0.324 |
| Intervals between OCT slices |  |  | 0.754 |
| 0.05-mm | 17 (8.4) | 3 (5.6) |  |
| 0.1-mm | 65 (32.2) | 19 (35.2) |  |
| 0.2-mm | 120 (59.4) | 32 (59.3) |  |

Values are mean ± SD, n (%), or median (25^th^-75^th^ percentile).

ACE-I = angiotensin-converting enzyme inhibitor; ACS = acute coronary syndromes; ARB = angiotensin II receptor blocker; CABG = coronary artery bypass graft; DAPT = dual anti-platelet therapy; eGFR = estimated glomerular filtration rate; HbA1c = hemoglobin A1c; HDL = high-density lipoprotein; LAD = Left anterior descending artery; LCX = Left circumflex artery; LDL = low-density lipoprotein; MI = myocardial infarction; MLA = Minimum lumen area; NSTEMI = non-ST-segment elevation myocardial infarction; OCT = optical coherence tomography; PCI = percutaneous coronary intervention; RCA = right coronary artery; SAP = stable angina pectoris; TCFA = thin-cap fibroatheroma; UAP = unstable angina pectoris; WBC = white blood cell.

**Supplementary Table S3. Comparison of MLA and intervals between OCT slices in ACS patients between plaque erosion and rupture**

| **Variables** | **ACS patients(n=256)** | | **P Value** |
| --- | --- | --- | --- |
|  | **Patients with plaque erosion (n=113, 44.1%)** | **Patients with plaque rupture (n=143, 55.9%)** |  |
| MLA measured by OCT | 1.01 (0.70-1.30) | 1.03 (0.87-1.39) | 0.099 |
| MLA measured by CTA | 1.08 (0.40-1.85) | 1.18 (0.69-1.89) | 0.244 |
| Intervals between OCT slices |  |  | 0.349 |
| 0.05-mm | 7 (6.2) | 13 (9.1) |  |
| 0.1-mm | 42 (37.2) | 42 (29.4) |  |
| 0.2-mm | 64 (56.6) | 88 (61.5) |  |

Values are n (%) or median (25^th^-75^th^ percentile).

Among 256 patients with ACS, MLA measured by OCT and CTA were calculated.

ACS = acute coronary syndromes; CTA = computed tomography angiography; MLA = Minimum lumen area; OCT = optical coherence tomography.

| **Supplementary Table S4. Comparison of diagnostic performances evaluated on ACS patients** | | | | | | |
| --- | --- | --- | --- | --- | --- | --- |
|  | **Sensitivity (%) (95% CI)** | **Specificity (%) (95% CI)** | **PPV (95% CI)** | **NPV (95% CI)** | **FPR (%) (95% CI)** | **FNR (%) (95% CI)** |
| **Patient-level diagnosis** | | | | | | |
| MD-CTA model | 83.9 (66.3-94.6) | 70.4 (49.8-86.3) | 76.5 (58.8-89.3) | 79.2 (57.9-92.9) | 29.6 (13.7-50.2) | 16.1 (5.4-33.7) |
| CNN model | 64.5 (45.4-80.8) | 66.7 (46.0-83.5) | 69.0 (49.2-84.7) | 62.1 (42.3-79.3) | 33.3 (16.5-54.0) | 35.5 (19.2- 54.6) |
| Reader 1 | 16.1 (5.5-33.7) | 70.4 (49.8-86.3) | 38.5 (13.9-68.4) | 44.2 (27.7-57.9) | 29.6 (13.7-50.2) | 83.9 (66.3-94.5) |
| Reader 2 | 12.9 (3.6-29.8) | 70.4 (49.8-86.3) | 33.3 (9.9-65.1) | 41.3 (27.0-56.8) | 29.6 (13.7-50.2) | 87.1 (70.2-96.4) |
| Reader 3 | 15.6 (5.3-32.8) | 63.0 (42.4-80.6) | 33.3 (11.8-61.6) | 38.6 (24.4-54.5) | 37.0 (19.4-57.6) | 84.4 (67.2-94.7) |
| **Slice-level diagnosis** | | | | | | |
| MD-CTA model | 74.5 (71.9-77.0) | 74.7 (73.4-76.1) | 45.3 (43.1-47.6) | 91.3 (90.2-92.2) | 25.3 (23.9-26.6) | 25.5 (23.0- 28.1) |
| CNN model | 62.6 (59.7-65.4) | 59.9 (58.4-61.4) | 30.5 (28.6-32.4) | 85.1 (83.7-86.3) | 40.1 (38.6-41.6) | 37.4 (34.6-40.3) |

| **Supplementary Table S5. Ablation studies on the key components of the Momentum Distillation-enhanced Composite Transformer Attention (MD-CTA) model** | | | | | | | |
| --- | --- | --- | --- | --- | --- | --- | --- |
|  | **AUC (95% CI)** | **Sensitivity (%) (95% CI)** | **Specificity (%) (95% CI)** | **PPV (%)**  **(95% CI)** | **NPV (%)**  **(95% CI)** | **FPR (%) (95% CI)** | **FNR (%) (95% CI)** |
| **Patient-level diagnosis** | | | | | | | |
| ViT without TL | 0.703 (0.600-0.806) | 67.7 (48.6-83.3) | 60.0 (48.0-71.2) | 41.2 (27.6-55.8) | 81.8 (69.1-90.9) | 40.0 (29.8-52.0) | 32.3 (16.7-51.4) |
| ViT + TL | 0.884 (0.823 - 0.945) | 80.7 (62.5 - 92.6) | 77.3 (66.2 - 86.2) | 59.5 (48.3 - 69.8) | 90.6 (82.3 - 95.3) | 22.7 (13.8 - 33.8) | 19.3 (7.4 - 37.5) |
| ViT + TL + ST | 0.872 (0.807 - 0.937) | 80.7 (62.5 - 92.6) | 78.7 (67.7 - 87.3) | 61.0 (49.5 - 71.4) | 90.8 (82.6 - 95.3) | 21.3 (12.7 - 32.3) | 19.3 (7.4 - 37.5) |
| ViT + TL + ST + MD (MD-CTA) | 0.899 (0.841 - 0.957) | 87.1 (70.2 - 96.4) | 85.3 (75.3 - 92.4) | 71.1 (58.3 - 81.2) | 94.1 (86.5 - 97.6) | 14.7 (7.6 - 24.7) | 12.9 (3.6 - 29.8) |
| **Slice-level diagnosis** | | | | | | | |
| ViT without TL | 0.773 (0.761-0.785) | 70.8 (68.1-73.5) | 69.5 (68.5-70.6) | 26.7 (25.2-28.3) | 93.8 (93.2-94.5) | 30.5 (29.4-31.5) | 29.2 (26.5-31.9) |
| ViT + TL | 0.886 (0.878 - 0.894) | 76.0 (73.4 - 78.4) | 80.8 (79.8 - 81.7) | 38.2 (36.9 - 39.6) | 95.6 (95.1 - 96.0) | 19.2 (18.2 - 20.2) | 24.0 (21.6 - 26.6) |
| ViT + TL + ST | 0.902 (0.895 - 0.909) | 81.0 (78.6 - 83.3) | 82.4 (81.5 - 83.2) | 41.9 (40.5 - 43.3) | 96.5 (96.1 - 96.9) | 17.6 (16.8 - 18.5) | 19.0 (16.7 - 21.4) |
| ViT + TL + ST + MD (MD-CTA) | 0.897 (0.890 - 0.904) | 82.2 (79.8 - 84.3) | 80.1 (79.1 - 81.0) | 39.3 (38.0 - 40.5) | 96.6 (96.2 - 97.0) | 19.9 (19.0 - 20.9) | 17.8 (15.7 - 20.2) |
| AUC = area under the curve; CI = confidence interval; PPV = positive predictive value; NPV = negative predictive value; FPR = false-positive rate; FNR = false-negative rate; ViT = vision transformer; TL = transfer learning; ST = sequential transformer; MD = momentum distillation-enhanced self-supervised pre-training. | | | | | | | |

| **Supplementary Table S6. Slice-level detection performances of the culprit lesion of the deep learning models** | | | | | | | |
| --- | --- | --- | --- | --- | --- | --- | --- |
|  | **AUC (95% CI)** | **Sensitivity (%) (95% CI)** | **Specificity (%) (95% CI)** | **PPV (95% CI)** | **NPV (95% CI)** | **FPR (%) (95% CI)** | **FNR (%) (95% CI)** |
| **Five-fold cross-validation** | | | | | | | |
| MD-CTA model | 0.935 (0.932-0.938) | 85.8 (85.4-86.2) | 85.5 (84.6-86.3) | 95.8 (95.5-96.0) | 61.1 (60.1-62.1) | 14.5 (13.7-15.4) | 14.2 (13.8-14.6) |
| CNN model | 0.693 (0.642-0.744) | 67.0 (66.4-67.6) | 73.9 (72.9-75.0) | 90.8 (90.4-91.2) | 36.9 (36.1-37.7) | 26.1 (25.0-27.1) | 33.0 (32.4-33.6) |
| **Test set validation** | | | | | | | |
| MD-CTA model | 0.929 (0.924-0.934) | 85.4 (84.5-86.2) | 83.3 (81.5-84.9) | 94.5 (93.9-95.1) | 62.6 (60.7-64.5) | 16.7 (15.1-18.5) | 14.6 (13.8-15.5) |
| CNN model | 0.796 (0.786-0.805) | 74.0 (72.9-75.1) | 68.7 (66.6-70.8) | 88.9 (88.1-89.8) | 43.8 (42.0-45.6) | 31.3 (29.2-33.4) | 26.0 (24.9-27.1) |
| AUC = area under the curve; CI = confidence interval; CNN = convolutional neural network; FNR = false-negative rate; FPR = false-positive rate; MD-CTA = momentum distillation-enhanced composite transformer attention; NPV = negative predictive value; PPV = positive predictive value. | | | | | | | |

| **Supplementary Table S7. Sub-analysis evaluating the discrimination performance between plaque erosion and plaque rupture** | | | | | | | | |
| --- | --- | --- | --- | --- | --- | --- | --- | --- |
|  | **AUC (95% CI)** | **Sensitivity (%) (95% CI)** | **Specificity (%) (95% CI)** | **PPV (95% CI)** | **NPV (95% CI)** | **FPR (%) (95% CI)** | **FNR (%) (95% CI)** |  |
| MD-CTA model | 0.708 (0.559 - 0.857) | 69.6 (47.1-86.8) | 67.7 (48.6-83.3) | 61.5 (47.3-74.0) | 75.0 (60.7-85.4) | 32.3 (16.7-51.4) | 30.4 (13.2-52.9) |  |
| CNN model | 0.585 (0.429-0.741) | 52.2 (30.6-73.2) | 51.6 (33.1-69.9) | 44.4 (31.9-57.7) | 59.3 (45.7-71.5) | 48.4 (30.1-66.9) | 47.8 (26.8-69.4) |  |
| AUC = area under the curve; CI = confidence interval; CNN = convolutional neural network; FNR = false-negative rate; FPR = false-positive rate; MD-CTA = momentum distillation-enhanced composite transformer attention; NPV = negative predictive value; PPV = positive predictive value. | | | | | | | | |

**Supplementary Figure S1. Flow diagram of the study**

**
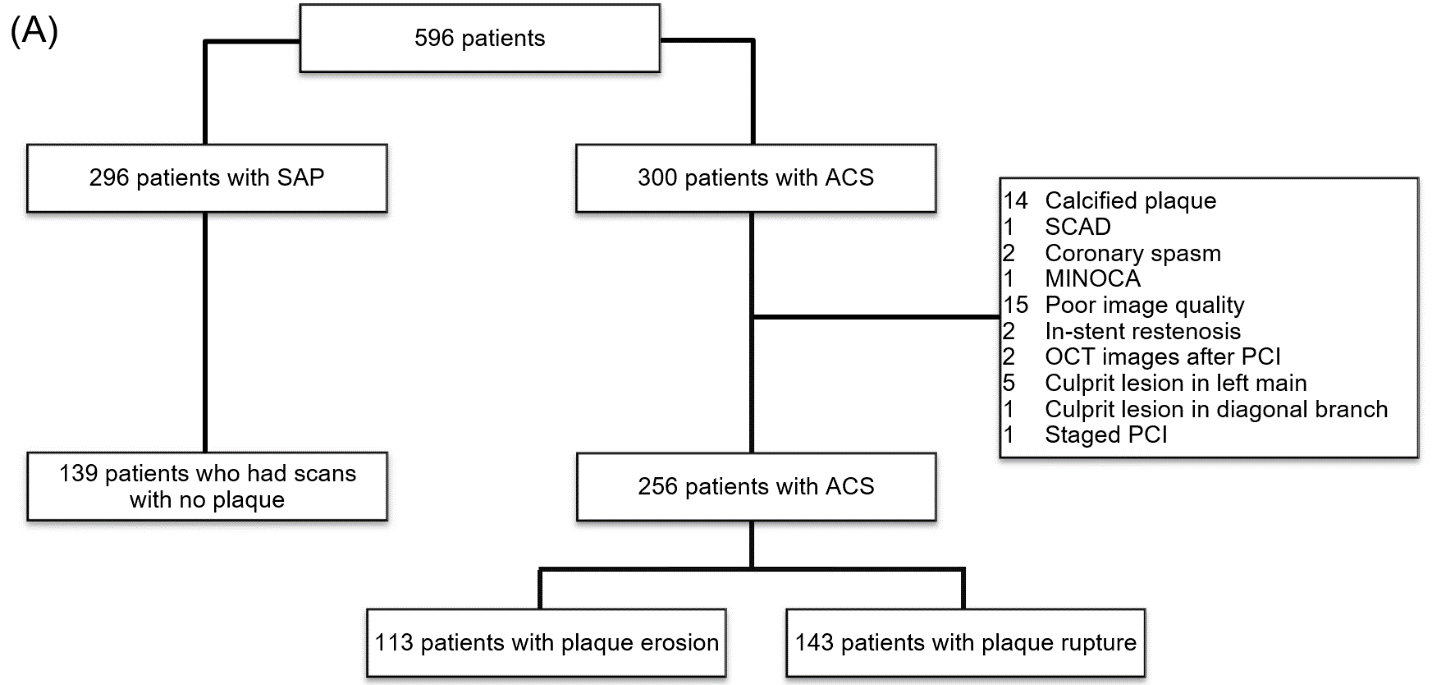
**


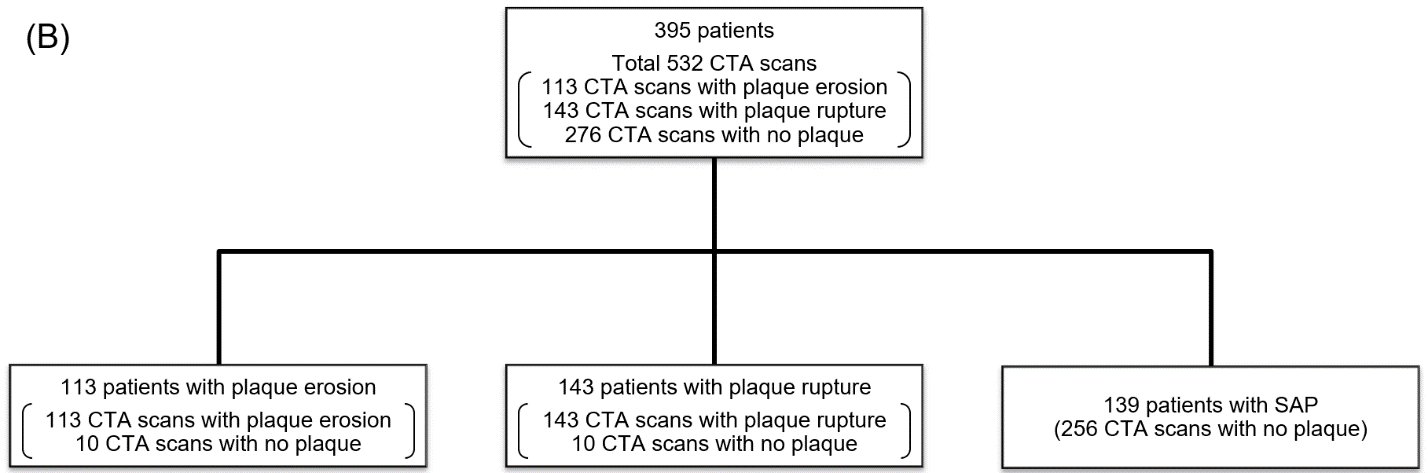


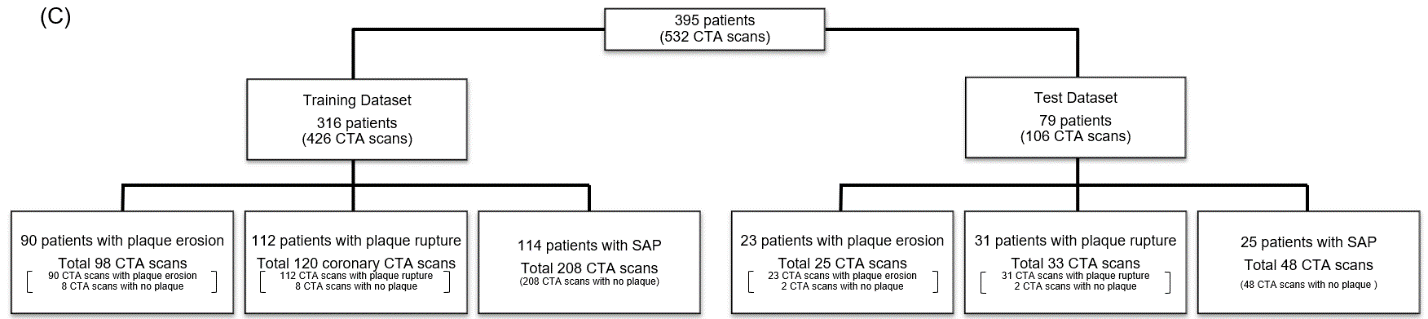


1. A total of 300 patients presenting with ACS and 296 with SAP underwent both CTA and OCT imaging before intervention between January 2011 and September 2022. Among ACS patients, 14 patients were excluded for calcified plaque, 1 for SCAD, 2 for coronary spasm, and 1 for MINOCA. In addition, 15 patients were excluded for poor image quality, 2 for in-stent restenosis, 2 for the absence of OCT images before PCI, 5 culprit lesions located in the left main, 1 for culprit lesion located in the diagonal branch, and 1 for staged PCI. Among the SAP patients, 139 patients with no plaque of > 10 mm in length were included. Thus, 256 ACS (113 with plaque erosion, 113 with rupture) and 139 SAP patients were included in the final analysis
2. Among 532 CTA scans, 276 CTA scans were from patients with ACS (113 CTA scans with plaque erosion, 143 with plaque rupture, and 20 with no plaque), and 256 scans with no plaque were from patients with SAP.
3. Among 256 ACS patients, the diagnosis of plaque erosion (n=113) or rupture (n=143) from the OCT images was used as the ground truth to determine pathology, and the site on the CTA image that matched the culprit plaque on that OCT image was determined to be the culprit lesion. Thus, there were 113 CTA scans with plaque erosion and 143 CTA scans with plaque rupture. In addition, CTA scans with no plaque were identified in patients with ACS (10 from plaque erosion and 10 from plaque rupture). The other 256 coronary CTA scans with no plaque were selected from 139 patients with SAP. Thus, a total number of 276 CTA scans were included as images with no plaque.

ACS = acute coronary syndrome; SCAD = spontaneous coronary artery dissection; CTA = computed tomography angiography; MINOCA = myocardial infarction with non-obstructive coronary artery; OCT = optical coherence tomography; PCI = percutaneous coronary intervention.

**Supplementary Figure S2. Details of the dataset division for the study**


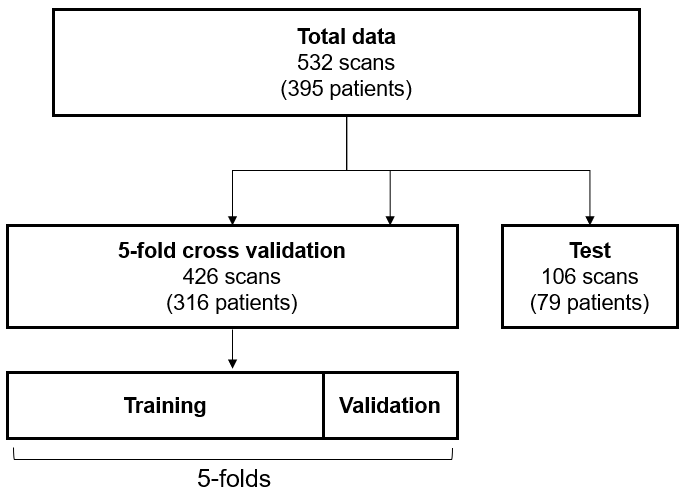


Among 532 scans from 395 patients, the data were divided into non-overlapping patient subsets. Specifically, 426 scans from 316 patients were used for five-fold cross-validation, and 106 scans from 79 patients were utilized for the test set validation.

**Supplementary Figure S3. Architecture of the Momentum Distillation-enhanced Composite Transformer Attention (MD-CTA) model** **
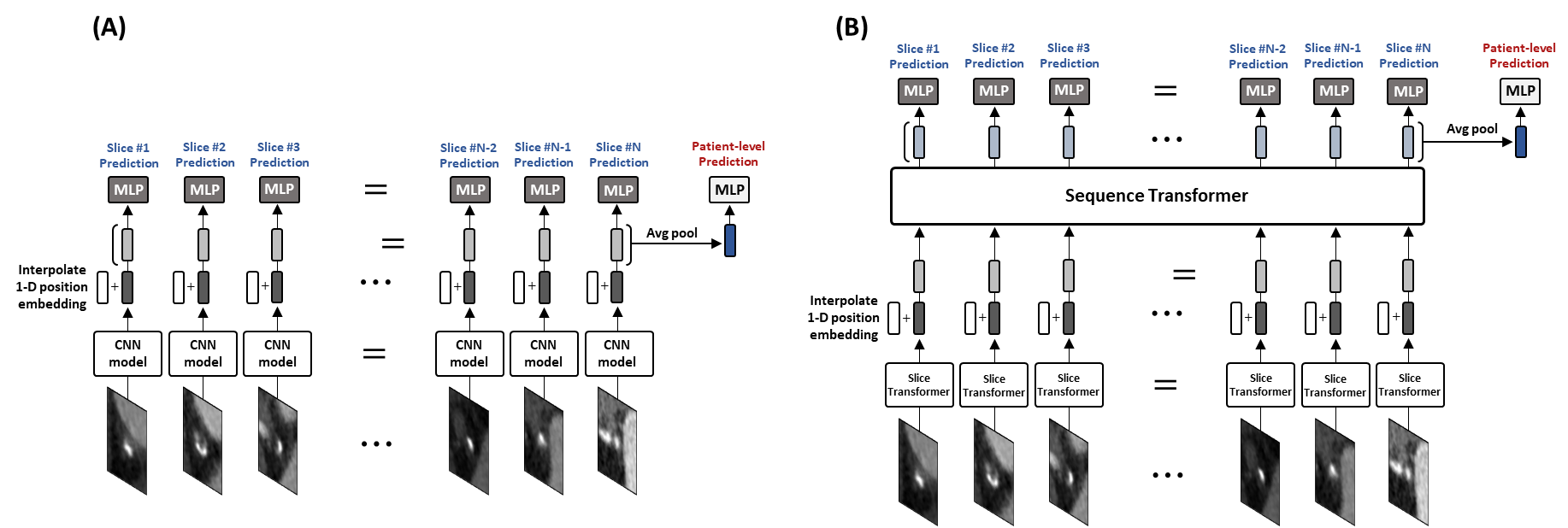
**

The proposed MD-CTA model leverages sequential as well as spatial attention to incorporate the information of the entire series of slices within the coronary computed tomography angiography scan (B), which is not possible for the standard convolutional neural network (CNN) model (A).

**Supplementary Figure S4. Training strategy of the Momentum Distillation-enhanced Composite Transformer Attention (MD-CTA) model**

**
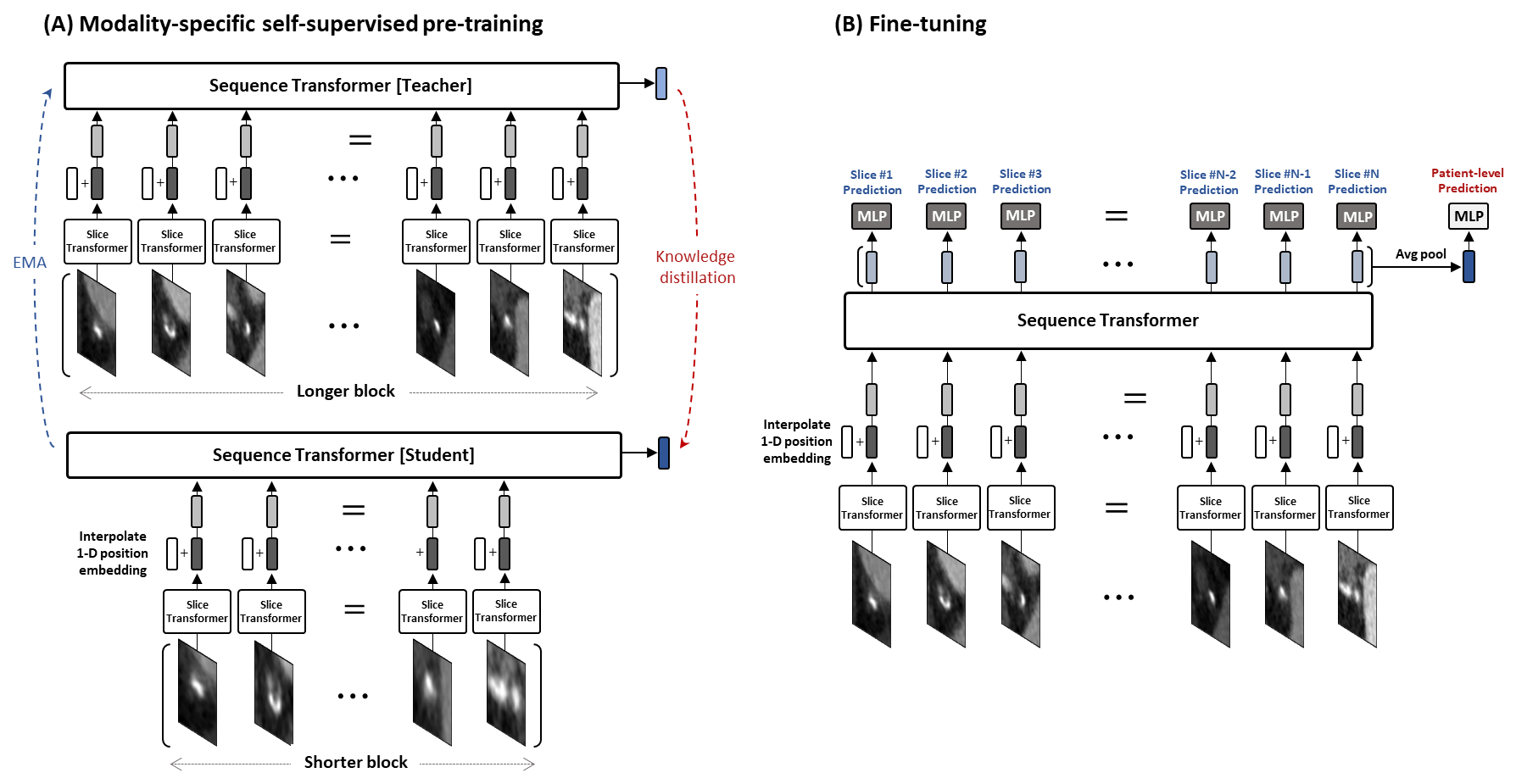
**

The MD-CTA model was first pre-trained with the momentum distillation-enhanced self-supervised learning (A), and subsequently fine-tuned to optimize patient-level and slice-level predictions simultaneously (B). This step-wise strategy significantly improved the overall performance of the model.

**Supplementary Figure S5. Confusion matrices of the model**


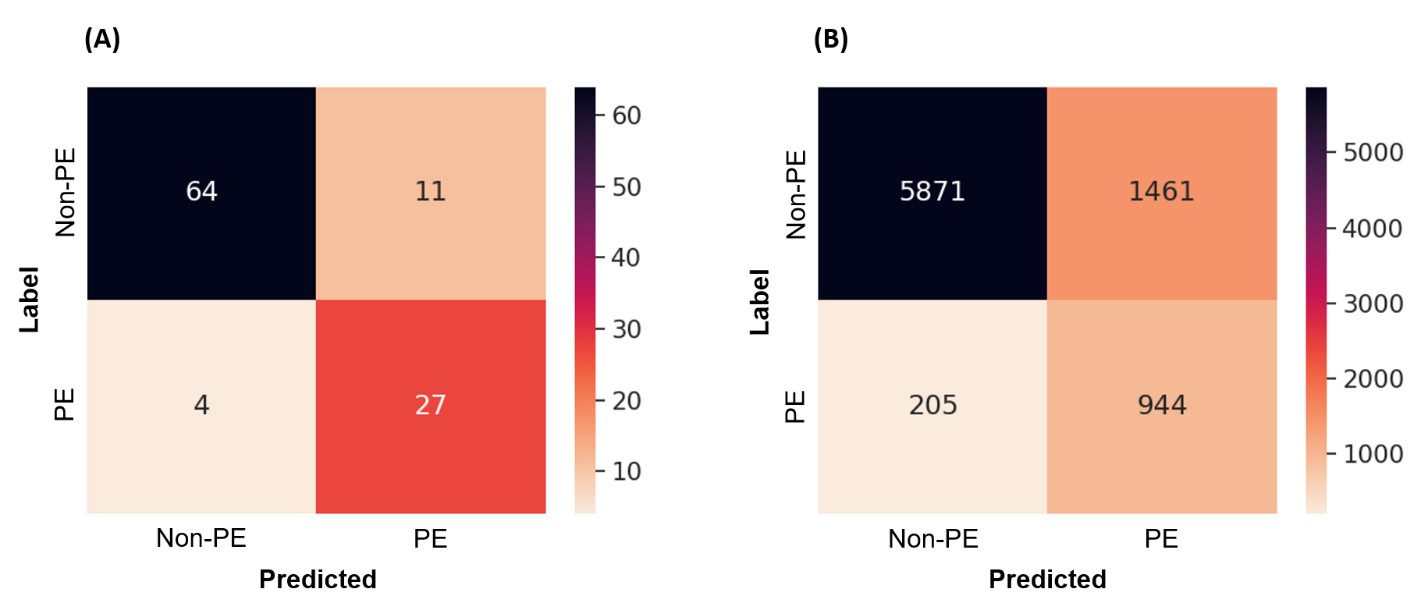


Confusion matrices of the Momentum Distillation-enhanced Composite Transformer Attention model in the test set validation are shown, both at the patient level (A) and slice level (B).

PE= plaque erosion.

**Supplementary Figure S6. Representative images of plaque rupture and plaque erosion as seen on OCT, CTA, and** **CTA enhanced by DL model**

**
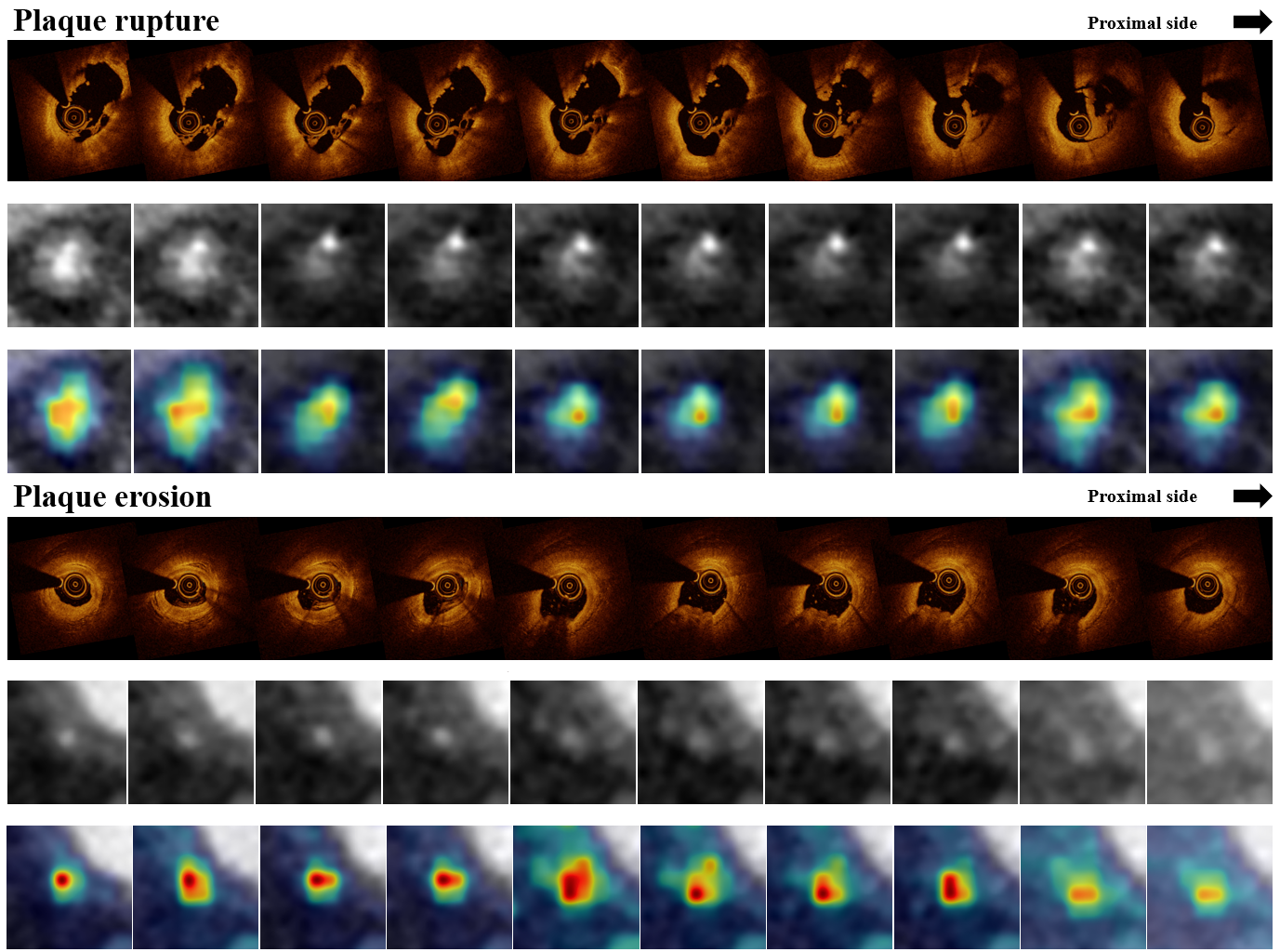
**

The OCT images of plaque rupture show the presence of fibrous cap discontinuity with a cavity formation within the plaque. The CTA images of plaque rupture show ruptured cap protruding into the vessel lumen at the same site observed by OCT. The CTA images enhanced by DL model show that the DL model specifically focuses on the ruptured cap.
The OCT images of plaque erosion show the presence of attached thrombus overlying an intact plaque. The CTA images of plaque erosion show a structure protruding into the vessel lumen at the site of attached thrombus observed by OCT. The CTA images enhanced by DL model show that the attention of the DL model has shifted from the narrowing of the lumen to the thrombus protruding into the lumen as the thrombus appears.

CTA = computed tomography angiography; DL = deep learning; OCT = optical coherence tomography.

**References**

1 Ruder, S. An overview of gradient descent optimization algorithms. *arXiv preprint arXiv:1609.04747* (2016).

2 Yamashita, R., Nishio, M., Do, R. K. G. & Togashi, K. Convolutional neural networks: an overview and application in radiology. *Insights into imaging* 9, 611-629 (2018).

3 Vaswani, A. *et al.* Attention is all you need. *Advances in neural information processing systems* 30 (2017).

4 Dosovitskiy, A. *et al.* An image is worth 16x16 words: Transformers for image recognition at scale. *arXiv preprint arXiv:2010.11929* (2020).

5 Naseer, M. M. *et al.* Intriguing properties of vision transformers. *Advances in Neural Information Processing Systems* 34, 23296-23308 (2021).

6 Park, N. & Kim, S. How Do Vision Transformers Work? *arXiv preprint arXiv:2202.06709* (2022).

7 Caron, M. *et al.* in *Proceedings of the IEEE/CVF International Conference on Computer Vision.* 9650-9660.

8 Pan, S. J. & Yang, Q. A survey on transfer learning. *IEEE Transactions on knowledge and data engineering* 22, 1345-1359 (2010).

9 Liu, Y. *et al.* Efficient training of visual transformers with small datasets. *Advances in Neural Information Processing Systems* 34, 23818-23830 (2021).

10 Jaiswal, A., Babu, A. R., Zadeh, M. Z., Banerjee, D. & Makedon, F. A survey on contrastive self-supervised learning. *Technologies* 9, 2 (2020).

11 Araki, M. *et al.* Diagnosis of coronary layered plaque by deep learning. *Sci Rep* 13, 2432, doi:10.1038/s41598-023-29293-6 (2023).
